# Development of the Ethiopian Healthy Eating Index (Et-HEI) and evaluation in women of reproductive age

**DOI:** 10.1017/jns.2022.120

**Published:** 2023-01-23

**Authors:** Tesfaye Hailu Bekele, Jeanne H. M. de Vries, Edith J. M. Feskens, Anneloes de Weijer, Inge D. Brouwer, Namukolo Covic, Laura Trijsburg

**Affiliations:** 1 Ethiopian Public Health Institute, Addis Ababa, Ethiopia; 2 Division of Human Nutrition and Health, Wageningen University, Wageningen, the Netherlands; 3 International Food Policy Research Institute, Addis Ababa, Ethiopia

**Keywords:** Diet quality, Dietary guidelines, Dietary index, Healthy diet

## Abstract

Ethiopia announced its first food-based dietary guidelines (FBDGs) on 15 March 2022. The present study aims to develop and evaluate the Ethiopian Healthy Eating Index (Et-HEI) based on the FBDG. Data were collected from 494 Ethiopian women of reproductive age sampled from households in five different regions. The Et-HEI consists of eleven components, and each component was scored between 0 and 10 points, the total score ranging from 0 to 110, with maximum adherence to the FBDG. The Et-HEI score was evaluated against the Minimum Dietary Diversity for Women (MDD-W) and the probability of nutrient adequacy. The average Et-HEI score for women of reproductive age was 49 out of 110. Adherence to the recommendations for grains, vegetables, legumes, fat and oils, salt, sugar and alcohol contributed the most to this score. Most women had low scores for fruits, nuts and seeds, and animal-sourced foods, indicating low intake. The Cronbach's alpha coefficient, indicating the reliability of the Et-HEI to assess its diet quality, was 0⋅53. The low mean Et-HEI score agreed with a low mean score of the MDD-W (3⋅5 out of 10). Also, low nutrient adequacies confirmed poor adherence to nutrient-dense components of the FBDG. The Et-HEI was not associated with the intake of vitamin B12, vitamin C and calcium in this study population. Women who completed secondary school and above had relatively lower Et-HEI scores. The newly developed Et-HEI is able to estimate nutrient adequacy while also assessing adherence to the Ethiopian FBDG though there is room for improvement.

## Introduction

**Fig. 1.**
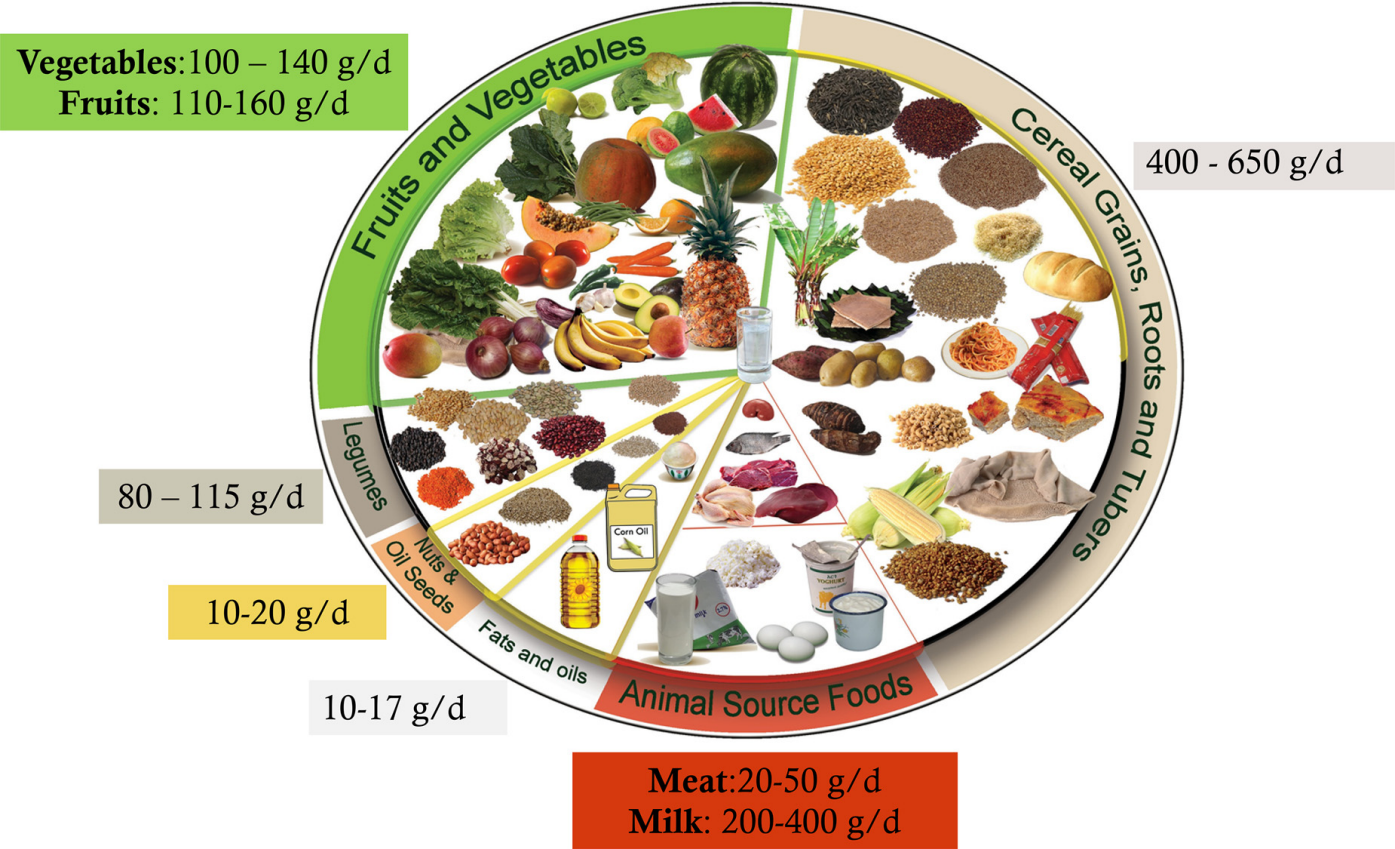
Ethiopian food guide, healthy plate (in Amharic Teenama MaĒD).

As part of the 2018 food and nutrition policy, the Ethiopian Government started developing the first food-based dietary guidelines (FBDGs) in collaboration with international and local partners^(1)^. The Ethiopian FBDG comprises eleven messages based on scientific evidence, i.e. to consume sufficient but not excessive amounts of whole grains; fruits and vegetables; milk and dairy foods; salt; meat, fish and eggs; legumes, nuts and seeds; fats and oils; added sugars; non-alcoholic and alcoholic beverages. To arrive at these recommendations regarding food group and amount, extensive reviews and secondary data analyses of local and global evidence combined with diet optimisation were conducted (Fig. 1 and the description in Supplementary Table S1). The individual and population diet optimizations were performed using data from 24-h dietary recalls (24HDR) collected among Ethiopian women from November to December 2019 and from the 2011 National Food Consumption Survey^(2,3)^. The FBDG was launched in March 2022 as an entry point for the national food system roadmap to make healthy diets more accessible, affordable, available and desirable to Ethiopians.

Several global and country-specific indices are available to evaluate diet quality^(4)^. The global indices are usually based on global dietary recommendations, nutrient adequacy of the diet or the prevention of non-communicable diseases. The country-specific indices are usually based on national FBDGs to assess adherence to these recommendations. A country-specific index can be used to compare diet quality across different subpopulations, investigate dietary intake trends, and further study diet-disease relationships to inform public health interventions and policy actions^(5,6)^. To assess the impact of the Ethiopian FBDG, the Ethiopian Healthy Eating Index (Et-HEI) was developed^(1)^. The Et-HEI assesses diet quality by determining the population's adherence to the Ethiopian FBDG in the population above 2 years of age.

Each HEI has a unique total score based on the number of components and the ratings assigned to each component. The scores for each component show the adherence to dietary guidelines for each food group or specific foods or nutrients, combined to provide a total healthy eating score^(5,7)^. Besides dietary components, an HEI may include a non-dietary component such as physical activity or environmental sustainability. However, low- and middle-income countries (LMICs) data on lifestyle and environmental sustainability is frequently missing; thus, non-dietary components are usually not considered^(5,8)^. The score for each component is determined by comparing the amount of food consumed with the recommended intake. An HEI that estimates the amount against the recommended quantity is preferred over qualitative estimates, despite the challenges of measuring portion sizes in LMICs^(5)^.

The Et-HEI was compared to another score, the Minimum Dietary Diversity for Women (MDD-W), the most commonly used index to assess nutrient adequacy in Ethiopia and other LMICs^(9)^. The present study aimed to define the Et-HEI based on the 2022 Ethiopian FBDG and to evaluate it against the probability of nutrient adequacy and the MDD-W in women of reproductive age (15–49 years).

## Methods

### Study design and participants

The Et-HEI score was calculated using two non-consecutive 24HDR, collected from 494 women of reproductive age in November and December 2019, residents of four out of eleven of Ethiopia's regions (Amhara, Oromia, Tigray and Southern Nation Nationalities) and the capital city (Addis Ababa). Data were collected in two districts from each region, one district with surplus agriculture production and one district with relatively low agriculture production, based on government agriculture performance measurements in 2017/2018. Additionally, two districts from Addis Ababa, one in high socio-economic and one in urban slum areas, were selected. The data collectors selected fifty households in each of the ten districts using a systematic random sampling method from newly listed households. One woman of reproductive age (household mother) from each selected household participated in the study. Six of the 500 women had incomplete data on dietary intake and were thus excluded from the final data analysis. Before the data collection started, the data collectors and field supervisors received one week of theoretical and practical training.

Study participants were asked about their age, religion, school attendance, level of education, family size, land size, the main source of drinking water and information about their house. The household food insecurity experience scale (FIES) was used to determine the extent the household experienced food insecurity in the past 12 months^(10)^. Then a quantitative multiple-pass 24HDR was conducted twice in women of reproductive age^(11)^. To improve the amount estimation of ingredients, all ingredients were measured on a digital food weighing scale after measuring a known weight. Every food item was measured twice to check the accuracy of the measurements. Substitutes, such as water, were weighed and converted using conversion factors if an ingredient was unavailable or impossible to measure. Standard portion sizes were used to determine the amount of ingredients when measuring the actual food was not possible or appropriate. Additionally, the data collectors measured height and weight of study participants using height boards to the nearest 0⋅1 cm and weighing scales to the nearest 0⋅1 kg. BMI was calculated from height and weight data.

Body composition was measured using Bodystat 1500 (bioelectrical impedance analysis device) to calculate the fat-free mass index (FFMI). FFMI (kg/m^2^) was calculated from the weight of fat-free mass (kg) by the height squared (m^2^). The body measurement devices were calibrated daily for a precise estimate. Before measurements, women were asked to remove all metal accessories and electronic devices. Measurements were taken within 10 min after the reading was begun to ensure consistency because the change in body position or movement would also affect the readings.

After the data collection, the data were checked for missing values. The data on conversion and edible factors of the foods were collected during 24HDR data collection, and raw-to-cooked factors were taken from Ethiopia's 2011 National Food Consumption Survey. The data were linked to the Ethiopia food composition table, revised during the 2011 National Food Consumption Survey. Finally, the food intake was converted into the daily intake of energy, protein, fat, carbohydrates, dietary fibre, calcium, iron, zinc, retinol, β-carotene, vitamin B1, vitamin B2, vitamin B6, vitamin B12, vitamin C, folate, vitamin B3 and vitamin A, determined in the form of retinol activity equivalent (RAE) using Compl-eat^(12)^.

### Developing the Et-HEI

**Fig. 2.**
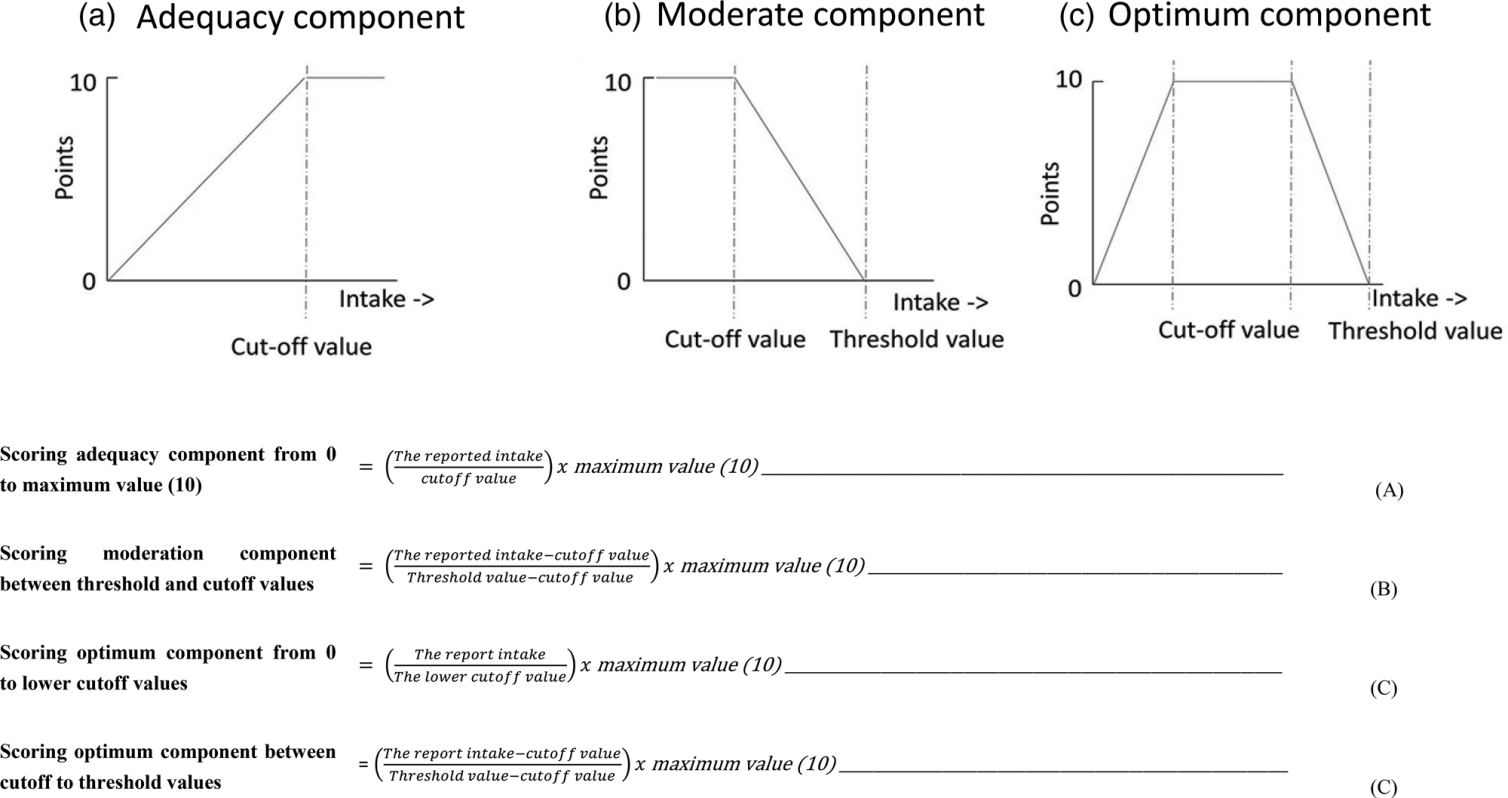
The formulas and graphic presentation of scoring for the Et-HEI for the different components: adequacy component (a), moderation component (b) and optimum component (c).

The Et-HEI, based on the Ethiopian FBDG, has eleven dietary components and one component on physical activity (Table 1). Depending on the type of recommendation, three types of scoring were used (A. adequate, B. moderate and C. optimum) as indicated in Table 1 and Fig. 2. Whole grains, roots and tubers, vegetables, fruits, legumes, nuts and seeds, and physical activity are adequacy components. A higher intake of these components reduces the risk of micronutrient deficiencies or chronic diseases such as type 2 diabetes and cardiovascular disease^(13)^. The consumption of added sugars and sugar-sweetened beverages (SSB), salt and alcohol should be limited due to their adverse health effects and were therefore categorised as moderation components^(13)^. Milk and dairy foods, meat, fish and eggs were categorised as optimum components as they may have both positive and negative health risks, depending on the amount consumed. For example, meat is a good source of iron and vitamin B12, but red and processed meat consumption is negatively associated with chronic diseases^(13)^. The recommended intakes for the eleven dietary components for the general population are based on an individual diet optimisation of women of reproductive age and population diet optimisation combined with additional local evidence and expert opinions^(14)^. Amore extensive description of the eleven dietary components is presented in Supplementary Table S1. In the present study, physical activity was not evaluated as data on the level of physical activity was not available and the focus was on diet quality.

**Table 1.** Et-HEI components with their classification for healthiness, type of scoring and recommended values in g/d for the population groups of 2 years of age and older, as well as minimum and maximum scores

No.	Dietary component	Relationship with health	Types of scoring	The amount for minimum score (0)	The amount for a maximum score (10)
1	Whole grains, roots and tubers	Protective	Adequacy	0	≥570
2	Vegetables	Protective	Adequacy	0	≥130
3	Fruits	Protective	Adequacy	0	≥150
4	Milk and dairy foods	Protective	Optimum	0 or ≥600	200–400
5	Meat, fish and egg	Protective	Optimum	0 or ≥70	20–50
6	Legumes	Protective	Adequacy	0	≥90
7	Nuts and seeds	Protective	Adequacy	0	≥15
8	Fats and oils	Limit	Optimum	0 or ≥27	10–17
9	Added sugar and SSB	Limit	Moderation	≥60	0–30
10	Salt	Limit	Moderation	≥8	0–5
11	Alcohol	Limit	Moderation	≥150	0–50
12	Physical activity^a^	Protective	Adequacy	0	≥3

a Moderate physical in number of days/week at least for 30 min.

SSB, sugar-sweetened beverage.

### Description of the Et-HEI components

#### The Et-HEI scoring

As all components are assumed to be equally important, they were similarly scored from 0 (minimum) to 10 (maximum), depending on the adherence to the FBDG. The summing of the separate scores resulted in a total score between 0 (no adherence to the guidelines) and 110 (maximal adherence to the guidelines). The following formulas for the different components were used, as indicated in Fig. 2.

### Evaluation of the Et-HEI by comparing it with the MDD-W

The MDD-W is a dichotomous dietary quality indicator developed for women of reproductive age (15–49 years) and is related to the micronutrient adequacy of the diet^(9,15)^. It assesses the consumption of ten food groups consumed during the last 24 h: starchy staples, beans and peas, nuts and seeds, dairy foods, flesh foods, eggs, vitamin A-rich dark green leafy vegetables, other vitamin A-rich vegetables and fruits, other vegetables and other fruits. Women consuming foods from five or more food groups are more likely to meet their micronutrient needs. Although the intake is assessed individually, the indicator is designed for population-level dietary assessments. The MDD-W is valid for predicting the adequacy of micronutrient intakes and is widely used, but NCD risk is not strongly correlated with the score^(16)^. The two 24HDRs were also used to estimate the MDD-W score. For this purpose, the consumed food items were grouped into ten food groups of the MDD-W. The contribution to each food category was identified based on local recipes for dishes containing several food groups. If these recipes were unavailable, the grouping was made using a similar local dish's recipe. The MDD-W score was then computed for each participant depending on how many food categories they consumed out of the ten food groups.

### Statistical analysis

Intakes of energy, nutrients and food groups were averaged over the two collection days. For each participant in the sample, the probability of adequacy was estimated for the eleven selected micronutrients (vitamin A, vitamin B1, vitamin B2, vitamin B3, folate, vitamin B6, vitamin B12, vitamin C, calcium, iron and zinc) taking their age category into account. The mean probability of adequacy (MPA) of all eleven micronutrients was calculated. Allen *et al.*^(17)^ provided estimated average requirements (EARs) for the age groups for the various nutrients, and CVs from Allen *et al.*^(18)^ were used to compute the standard deviation (sd) using the formula: sd is equal to CV*EAR/100. The Wiesmann *et al.* (2009) approach, described in Table A6-2^(19)^, was applied to calculate the probability of iron adequacy for women of reproductive age. For example, the probability of adequacy will be 0 if the usual iron intake in adult women is <15⋅91 mg/d (5 % bioavailability).

The Et-HEI was assumed to provide enough variation in scores to detect a meaningful difference in diet quality and adherence to the dietary guidelines in the population like other indexes with a similar purpose^(20,21)^. Energy, carbohydrate, protein and fat intake were reported using a median, and selected micronutrient intakes reported the probability of adequacy. The association of age, BMI, FFMI, the probability of adequacy for each nutrient, and MPA across the Et-HEI and MDD-W categories were analysed using regression and robust standard error. ANCOVA was used to adjust for energy intake across quartiles of Et-HEI and MDD-W. In addition, the percentage of carbohydrate and nutrient density per 1800 kcal across Et-HEI was estimated (Supplementary Table S2). For the categorical variables household food insecurity experience scale (HFIES) and level of education, the differences between dietary index quartiles were tested using Kendall's tau statistic. The association between the total score of Et-HEI and MDD-W and between their corresponding components was analysed using Kendall's tau statistic. To check the internal consistency of the different components to assess total diet quality, we estimated Cronbach's coefficient *α*. Principal component analysis (PCA) was used to determine the number of components that determine most of the variation. All statistical analyses were carried out with STATA version 14.2, using a *P*-value of <0⋅05 for statistical significance.

## Results

### Characteristics of the study population

The average age of the study population was 31 years, and the majority had no (52 %) or primary (31 %) education. Most women (65 %) had a normal body weight (BMI: 18⋅5–25 kg/m^2^) or were underweight (23 % with BMI < 18⋅5 kg/m^2^). Mean ages did not differ between the Et-HEI quartiles (Table 2). BMI was lower in the highest Et-HEI quartiles, while women in secondary school and above scored more frequently in the lower Et-HEI quartiles. MDD-W score was higher in the highest quartiles of the Et-HEI score.

**Table 2.** Differences in characteristics across Et-HEI quartiles among women of reproductive age

Characteristics	*N*	Quartiles of Et-HEI				*P*-value*
		Q1 (*n* 124)	Q2 (*n* 123)	Q3 (*n* 124)	Q4 (*n* 123)	
Et-HEI, mean (sd)	494	37⋅5 (4⋅5)	46⋅2 (1⋅8)	51⋅5 (1⋅4)	59⋅9 (4⋅4)	–
Age (year), mean (sd)	489	32⋅6 (7⋅8)	32⋅3 (7⋅9)	31⋅3 (7⋅8)	31⋅0 (7⋅7)	0⋅522
BMI (kg/m^2^), mean (sd)	482	22⋅0 (4⋅5)	21⋅0 (3⋅7)	21⋅1 (3⋅7)	20⋅1 (3⋅0)	0⋅027
FFMI (kg/m^2^), mean (sd)	474	15⋅5 (1⋅7)	15⋅4 (1⋅7)	15⋅6 (1⋅6)	15⋅2 (1⋅7)	0⋅972
Level of education						0⋅011
No formal education, *n* (%)	254	53 (21)	60 (24)	69 (27)	72 (28)	
Primary education, *n* (%)	153	40 (26)	39 (25)	41 (27)	33 (22)	
Secondary education, *n* (%)	68	24 (35)	21 (31)	11 (16)	12 (18)	
Higher education, *n* (%)	14	7 (50)	3 (21)	3 (21)	1 (7)	
HFIES						0⋅742
Secure, *n* (%)	131	38 (29)	33 (25)	31 (24)	29 (22)	
Mild insecure, *n* (%)	133	31 (23)	29 (22)	37 (28)	36 (27)	
Moderate insecure, *n* (%)	117	25 (21)	32 (27)	33 (28)	27 (23)	
Severe insecure, *n* (%)	108	30 (28)	29 (27)	23 (21)	26 (24)	
MDD-W, mean (sd)	494	3⋅3 (0⋅7)	3⋅2 (0⋅5)	3⋅4 (0⋅6)	3⋅7 (0⋅6)	<0⋅001

Et-HEI, Ethiopian Health Eating Index; sd, standard deviation; BMI, body mass index (number of women in Q1 = 122, Q2 = 121, Q3 = 122 and Q4 = 117), FFMI, fat-free mass index (Q1 = 121, Q2 = 119, Q3 = 120, Q4 = 114); HFIES, Household Food Insecurity Experience Scale; MDD-W, Minimum Dietary Diversity for Women.

* *P*-value from regression and estimated robust standard error.

**Fig. 3.**
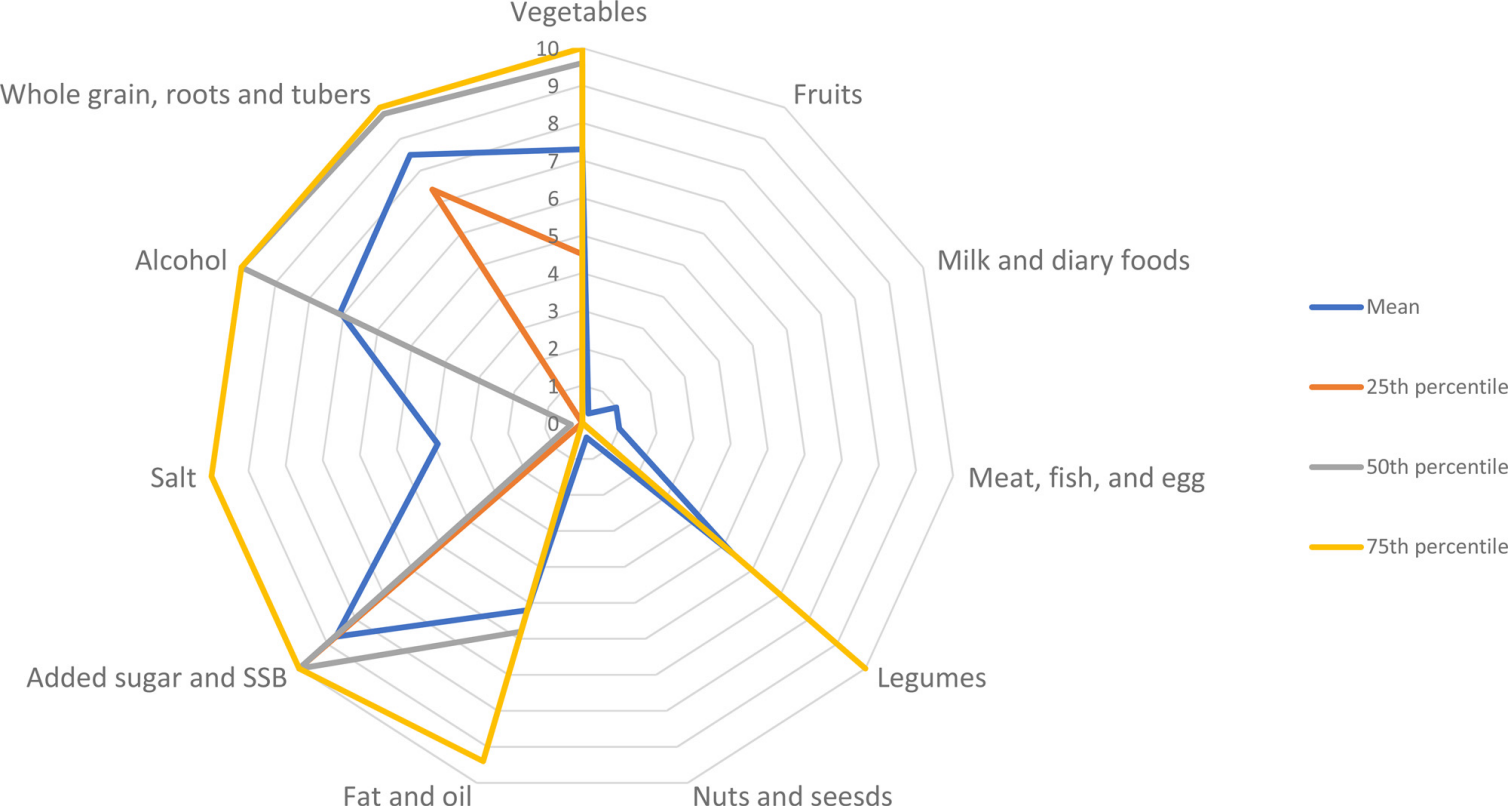
The scoring of 11 dietary components of the Et-HEI in women of reproductive age scored between 0 and 10 points.

The mean Et-HEI score was 49, ranging from 24 to 71 out of 110 points (see Supplementary Fig. S1). Women generally scored low mean points out of a maximum of 10 points for the components fruits (0⋅3), milk and dairy foods (1⋅0), meat, fish and eggs (1⋅0), and nuts and seeds (0⋅4) due to very low adherence to the recommended intake of these food groups. Conversely, most women scored much higher on the components of whole grain, root and tuber (8⋅5), vegetables (7⋅3), added sugar, and SSB (8⋅7), and alcohol consumption (7⋅1), showing a better adherence to these components than to the other components (Fig. 3). If the women consumed milk and dairy foods and meat, fish and eggs, they often met the recommended intake, but this was not the case if they consumed fruit, nuts and seeds. The Cronbach's coefficient value *α* = 0⋅53 indicated the internal consistency of the Et-HEI to measure overall diet quality.

The total Et-HEI and the components score did not show a correlation with the total energy consumption except for whole grain, roots and tubers (Supplementary Table S3). The multidimensionality (at least five dimensions) that underly the Et-HEI is demonstrated by the scree plot in the PCA-examining the Et-HEI (Supplementary Fig. S2). This finding indicated that the covariation in dietary habits cannot be described by a single linear combination of the Et-HEI component scores.

### Association of energy and macronutrient intake and micronutrients adequacy with the Et-HEI

Table 3 shows the mean energy, carbohydrate, protein and fat intake across the Et-HEI quartiles and the probability of adequately meeting micronutrient requirements for both unadjusted and energy-adjusted intakes among women. The median energy (2678 kcal/d), carbohydrate (467 g/d), protein (1⋅3 g/kg of body weight/d) and fat (41 g/d) were different between the Et-HEI quartiles. Carbohydrate and protein intake were positively associated with Et-HEI. After energy adjustment, carbohydrate and protein intake remained associated, whereas fat intake was inversely associated with Et-HEI. Vitamin B1, B6 and iron remained positively associated with ET-HEI after energy adjustments. Associations of Et-HEI with vitamin B2 and folate were reduced after energy adjustment. MPA was higher in the higher Et-HEI quartiles, especially before energy adjustment.

**Table 3.** Median energy, carbohydrate, protein and fat and probability of adequacy (PA) for nutrient intake across Et-HEI quartiles

	Median (*N* 494)	Quartiles of Et-HEI				*P*-value*
		Q1 (*n* 124)	Q2 (*n* 123)	Q3 (*n* 124)	Q4 (*n* 123)	
Energy (kcal/d)	2678⋅1	2581⋅2	2486⋅7	2779⋅6	2865⋅1	0⋅391
Unadjusted nutrient intake						
Carbohydrate (g/d)	466⋅9	440⋅1	431⋅1	489⋅0	507⋅5	0⋅038
Protein (g/kg/d)	1⋅3	1⋅2	1⋅2	1⋅3	1⋅5	0⋅023
Fat (g/d)	40⋅7	43⋅0	37⋅5	40⋅6	41⋅8	0⋅677
Micronutrients	PA					
Vitamin A (RAE, μg/d)	0⋅3	0⋅2	0⋅3	0⋅3	0⋅3	0⋅049
Vitamin B6 (mg/d)	0⋅9	0⋅8	0⋅8	0⋅9	0⋅9	0⋅003
Vitamin B12 (ug/d)	0⋅0	0⋅0	0⋅0	0⋅0	0⋅0	0⋅724
Vitamin C (mg/d)	0⋅1	0⋅1	0⋅1	0⋅1	0⋅0	0⋅065
Calcium (mg/d)	0⋅3	0⋅3	0⋅3	0⋅3	0⋅3	0⋅665
Folate (ug/d)	0⋅8	0⋅8	0⋅8	0⋅8	0⋅9	0⋅034
Iron (mg/d)	0⋅9	0⋅8	0⋅9	0⋅9	0⋅9	<0⋅001
Vitamin B3 (mg/d)	0⋅6	0⋅6	0⋅6	0⋅7	0⋅7	0⋅679
Vitamin B2 (mg/d)	0⋅6	0⋅5	0⋅5	0⋅6	0⋅6	0⋅738
Vitamin B1 (mg/d)	0⋅8	0⋅8	0⋅8	0⋅9	0⋅9	<0⋅001
Zinc (mg/d)	1⋅0	1⋅0	1⋅0	1⋅0	1⋅0	0⋅493
MPA	0⋅6	0⋅5	0⋅5	0⋅6	0⋅6	0⋅018
Energy-adjusted nutrient intake						
Carbohydrate (g/d)		457⋅1	464⋅7	471⋅2	474⋅6	<0⋅001
Protein (g/kg/d)		1⋅2	1⋅3	1⋅3	1⋅4	0⋅002
Fat (g/d)		44⋅1	39⋅7	39⋅4	39⋅7	0⋅021
Micronutrients						
Vitamin A (RAE, μg/d)		0⋅2	0⋅3	0⋅3	0⋅3	0⋅283
Vitamin B6 (mg/d)		0⋅8	0⋅8	0⋅9	0⋅9	0⋅037
Vitamin B12 (ug/d)		0⋅0	0⋅0	0⋅0	0⋅0	0⋅575
Vitamin C (mg/d)		0⋅1	0⋅1	0⋅1	0⋅0	0⋅083
Calcium (mg/d)		0⋅3	0⋅3	0⋅3	0⋅3	0⋅854
Folate (ug/d)		0⋅8	0⋅8	0⋅8	0⋅8	0⋅218
Iron (mg/d)		0⋅9	0⋅9	0⋅9	0⋅9	0⋅005
Vitamin B3 (mg/d)		0⋅7	0⋅6	0⋅6	0⋅7	0⋅241
Vitamin B2 (mg/d)		0⋅5	0⋅6	0⋅5	0⋅6	0⋅426
Vitamin B1 (mg/d)		0⋅8	0⋅8	0⋅9	0⋅9	0⋅002
Zinc (mg/d)		1⋅0	1⋅0	1⋅0	1⋅0	0⋅897
MPA		0⋅6	0⋅6	0⋅6	0⋅6	0⋅031

Et-HEI, Ethiopian Health Eating Index; MPA, mean probability of adequacy; RAE, retinol activity equivalent.

* *P*-value from regression and estimated robust standard error.

The MPA was high for vitamin B6 (0⋅9), folate (0⋅8), iron (0⋅9), vitamin B1 (0⋅8) and zinc (1⋅0), but much lower for vitamin A (0⋅3), vitamin B12 (0⋅0), vitamin C (0⋅1) and calcium (0⋅3). The MPA was 0⋅5 in the first and second, and 0⋅6 in the third and fourth Et-HEI quartiles of the eleven micronutrients.

For comparison, the mean macro and micronutrients across quartiles of the MDD-W were analysed (Table 4). MDD-W was a positive association with daily energy intake. After adjusting for energy intake, carbohydrate and fat intake were positively associated with MDD-W. However, protein intake per kg body weight was not different across quartiles of MDD-W. Vitamin A, B12 and iron were all positively associated with MDD-W before and after adjusting energy intake. After adjusting for energy intake, the positive associations of vitamin B1, B3, B6, zinc and calcium with MDD-W disappeared. MPA based on the eleven micronutrients was strongly associated with MDD-W.

**Table 4.** Median energy, carbohydrate, fat and protein intake and probability of adequate nutrient intake across MDD-W quartiles

	Quartiles of MDD-W				*P*-value*
	Q1 (*n* 209)	Q2 (*n* 151)	Q3 (*n* 93)	Q4 (*n* 41)	
Energy (kcal)	2562⋅0	2659⋅6	2936⋅4	2752⋅3	0⋅001
Unadjusted nutrient intake					
Carbohydrate (g/d)	454⋅9	460⋅4	510⋅7	452⋅4	0⋅033
Protein (g/kg/d)	1⋅2	1⋅3	1⋅4	1⋅3	0⋅005
Fat (g/d)	35⋅5	40⋅9	47⋅0	53⋅5	<0⋅001
Micronutrients					
Vitamin A (RAE, μg/d)	0⋅2	0⋅3	0⋅5	0⋅4	<0⋅001
Vitamin B6 (mg/d)	0⋅8	0⋅9	0⋅9	0⋅9	<0⋅001
Vitamin B12 (ug/d)	0⋅0	0⋅0	0⋅1	0⋅2	<0⋅001
Vitamin C (mg/d)	0⋅1	0⋅1	0⋅1	0⋅1	0⋅125
Calcium (mg/d)	0⋅3	0⋅3	0⋅4	0⋅5	0⋅001
Folate (ug/d)	0⋅8	0⋅8	0⋅9	0⋅9	0⋅059
Iron (mg/d)	0⋅8	0⋅9	0⋅9	0⋅9	<0⋅001
Vitamin B3 (mg/d)	0⋅6	0⋅7	0⋅8	0⋅7	<0⋅001
Vitamin B2 (mg/d)	0⋅5	0⋅5	0⋅6	0⋅6	0⋅370
Vitamin B1 (mg/d)	0⋅8	0⋅8	0⋅9	0⋅9	0⋅038
Zinc (mg/d)	1⋅0	1⋅0	1⋅0	1⋅0	0⋅023
MPA	0⋅5	0⋅6	0⋅6	0⋅6	<0⋅001
Energy-adjusted nutrient intake					
Carbohydrate (g/d)	475⋅5	463⋅7	465⋅0	439⋅2	<0⋅001
Protein (g/kg/d)	1⋅3	1⋅3	1⋅3	1⋅3	0⋅128
Fat (g/d)	36⋅8	41⋅1	43⋅8	52⋅7	<0⋅001
Micronutrients					
Vitamin A (RAE, μg/d)	0⋅2	0⋅3	0⋅5	0⋅4	<0⋅001
Vitamin B6 (mg/d)	0⋅8	0⋅9	0⋅9	0⋅9	0⋅170
Vitamin B12 (ug/d)	0⋅0	0⋅0	0⋅1	0⋅2	<0⋅001
Vitamin C (mg/d)	0⋅1	0⋅1	0⋅1	0⋅1	0⋅285
Calcium (mg/d)	0⋅3	0⋅3	0⋅3	0⋅4	0⋅115
Folate (ug/d)	0⋅8	0⋅8	0⋅8	0⋅8	0⋅610
Iron (mg/d)	0⋅9	0⋅9	0⋅9	0⋅9	0⋅032
Vitamin B3 (mg/d)	0⋅6	0⋅7	0⋅7	0⋅7	0⋅179
Vitamin B2 (mg/d)	0⋅6	0⋅5	0⋅5	0⋅6	0⋅612
Vitamin B1 (mg/d)	0⋅8	0⋅8	0⋅9	0⋅9	0⋅105
Zinc (mg/d)	1⋅0	1⋅0	1⋅0	1⋅0	0⋅471
MPA	0⋅5	0⋅6	0⋅6	0⋅6	<0⋅001

MPA, mean probability of adequacy; MDD-W, Minimum Dietary Diversity for Women; RAE, retinol activity equivalent.

* *P*-value from regression and estimated robust standard error.

The MDD-W score does not include fats, oils, added sugar, SSB, salt or alcohol. The Spearman correlation between the total score of Et-HEI and MDD-W was moderate (*r* = 0⋅30). The Kendall's tau correlation between the components, fruits (*τ* = 0⋅91), milk and dairy foods (*τ* = 0⋅94), and nuts and seeds (*τ* = 0⋅97) were high. Also, legumes (*τ* = 0⋅62) were strongly associated with Et-HEI and MDD-W (Supplementary Table S4).

## Discussion

The Et-HEI is expected to assess adherence to healthy dietary patterns, capture dietary diversity and nutrient adequacy, and reduce the risk of NCD similar to other HEI^(22,23)^. In Ethiopia, energy and nutrient adequacy remains key issue as most low-income people's diets are low in nutrient-dense foods and dominated by starchy staple foods^(24)^. The Et-HEI quantitatively evaluated how well an index estimates the diet quality based on selected nutrients and how comparable the performance was with MDD-W, indicating a need for further adjustment^(25)^. The Et-HEI tool measures diet quality independent of quantity as a very low (*r* = 0⋅1) correlation was observed between total Et-HEI score and energy intake (Supplementary Table S3) and a positive association with the mean probability of nutrient adequacy. However, when we checked the association of macro and micronutrient intake across the Et-HEI quartiles, we found an association with carbohydrates, fat, vitamin B12 and vitamin B1 intake which need further investigation with a larger study population.

We found a positive correlation between the Et-HEI and MDD-W, and both scores were relatively low. These low Et-HEI and dietary diversity scores agreed with the low probability of women's adequate vitamin A, vitamin B12, vitamin C and calcium intake. Similarly, we observed a positive association between the Et-HEI and MPA. However, there was no difference in the mean probability of adequate vitamin A, vitamin B12 and calcium intake between the Et-HEI quartiles before and after adjusting for energy intake. This might be due to the low intakes of vitamin A, vitamin B12 and calcium in our study population; mean vitamin A, vitamin B12 and calcium intake were about one-fifth of the EAR in our study, which agrees with previous study findings^(3,26)^. In contrast, MDD-W has been associated with unadjusted and energy-adjusted vitamin A intake; this may be because MDD-W includes a separate subgroup of dark green leafy vegetables rich in vitamin A^(25)^. Sub-dividing vegetables into dark green leafy vegetables and other vegetables in Et-HEI may improve the estimation of vitamin A adequacy as the Ethiopian diet is mostly plant-based^(27,28)^. The overall associations of micronutrients with MDD-W were stronger than for Et-HEI, while for macronutrients such as carbohydrates and protein more positive associations with Et-HEI than MDD-W were observed. In addition, the association between fat with Et-HEI was more negatively associated than with MDD-W. These findings indicate that Et-HEI takes into account dietary patterns related to NCDs besides nutrient adequacy which fits the purpose of the new FBDG in Ethiopia.

It was found that women with a higher BMI were more likely to be in the first and second quartile of Et-HEI, a finding replicated in research including the US-based HEI^(29)^. However, in our study, women with higher education were also more likely to be in the first and second quartiles, which was unexpected and needs further investigation. Additional analysis of the Et-HEI components showed that women who completed secondary school or above consumed relatively more vegetables, fruits, grains, and alcohol and salt, whereas dairy, meat, legumes and nuts were more consumed by women who did not attend formal education or completed primary school. The observation of higher socio-economic status being associated with healthier lifestyles and diets is most findings from high-income countries, while it has been reported before that rural or lower-educated subjects may have better diets than higher educated participants when studying low-income countries^(30)^. Living in urban areas without the opportunity to make healthy dietary choices may also lead to low HEI scores.

Women of reproductive age had a median Et-HEI of less than half of the maximum score with an IQR (25th–75th percentile) of 49 (43–54) out of 110. Ethiopian women's diets consisted primarily of grains, vegetables, legumes, fats and oils. Fruits, meat, fish, eggs, dairy, nuts and seeds were occasionally consumed. Low consumption of fruits, vegetables, nuts and seeds resembles an unbalanced diet and is associated with several non-communicable diseases related to increased mortality rates^(31,32)^. Maintaining the current intake of whole grains, roots, tubers, legumes and vegetables and the current practice of limiting added sugar, SBB and alcohol consumption must be encouraged. For better adherence to the Ethiopian FBDG, it is important to substantially increase the intake of fruits, milk, dairy foods, meat, fish, eggs, nuts and seeds. The lower Et-HEI score was explained by mainly consuming plant-based and less diversified food sources, similar to previous dietary assessment findings that indicated low dietary diversity in Ethiopia^(26,33)^. The Cronbach's coefficient value *α* = 0⋅53 indicated that the components of the Et-HEI have shared covariance and probably measure the same underlying concept, total diet quality. The PCA showed the multidimensional nature of the Et-HEI as the underlying construct of diet quality as Supplementary Fig. S2 showed five components have >1 eigenvalue^(15)^. This indicated that the Et-HEI's components score is equally important as the total score. However, the distribution of the component may not be wide enough to detect differences in diet quality among individuals as some of the components are barely consumed by study participants (Supplementary Fig. S1). Therefore, it is important to use the component's score along with the total Et-HEI score.

The inadequate nutrient intake reflects poor overall diet quality or low food group intake, which reflects on the Et-HEI score. For example, vitamin B12 is mainly found in animal-sourced foods^(34)^, and a low intake of animal-sourced food is considered a public health problem in Ethiopia because of inadequate nutrient intake^(26,35)^. For this reason, the consumption of red meat, fish, egg and dairy foods should be encouraged^(26,36)^, as recommended in the 2022 Ethiopian FBDG. Ethiopia's low consumption of fruits by the whole population is probably the cause of the low vitamin C intake (31⋅0 mg/d)^(37)^. Future research should evaluate vitamin C, vitamin A and calcium intake in women consuming high amounts of fruit and vegetables to investigate to what extent requirements can be met^(27,28)^.

According to the Et-HEI, obtaining the maximum score (110) is impossible if no animal-sourced foods are consumed. On average, 20–50 g/d of meat, fish and eggs should be consumed to receive a maximum of 10 points, and 200–400 g/d of milk and dairy foods should be consumed for another 10 points. The Ethiopian Orthodox Tewahedo Church has around 43⋅5 % followers in the population and practices up to 140 fasting days a year^(38)^. Fasting means avoiding animal-sourced foods or following a vegan diet and staying with no food and drinks for a certain time during the day throughout the fasting days. In the FBDG, this is accounted for in the recommendations by the advice to replace animal-sourced foods with legumes, nuts and seeds during fasting. According to the current Et-HEI, a fasting person could only obtain a maximum score of 90 points, even if good replacers are included in the diet. In the future, collecting dietary and related data during fasting should allow scoring of a different combination of food groups than in the current Et-HEI such as legumes, nuts and seeds different to better assess the diet quality and adherence to the FBDG during fasting. For example, the American HEI-2010 and HEI-2005 classify beans as protein or vegetables depending on whether they are the main protein source^(20)^.

Due to a lack of data and the study's focus on evaluating Et-HEI for diet quality, the physical activity component was not included in the Et-HEI. Other comparable evaluations do not consider physical activity for the same reason^(8,16,25)^. Nevertheless, physical activity is part of the Ethiopian FBDGs and Et-HEI because it is important to maintain energy balance and healthy body weight^(39)^.

Et-HEI showed no association with energy intake and MDD-W were weakly positively associated with energy intake. It may not be beneficial if the Et-HEI provided higher scores for people having simply a higher consumption of the unhealthy component of Et-HEI food groups because the goal of the guidelines is to improve diet quality for optimal health^(29)^ and to keep body weight stable. Besides the higher energy intake, the source, whether the extra energy comes from healthy or unhealthy components, and the dietary and non-dietary mitigation is appropriate. Therefore, the possibility of including the level of physical activity or energy expenditure to balance energy intake should be investigated further with the high-risk population for overweight.

Et-HEI is the first healthy eating index targeted at a specific country developed on the African continent based on FBDG. Only a few low and middle-income African countries have such an index^(5)^, mainly because they do not have FBDG^(40)^. In most African nations with FBDG, their dietary guidelines lack the recommended amount due to a lack of quantitative dietary intake data and expertise in diet optimisation. Therefore, the findings of the present study will serve as an important example for other LMICs, particularly in Africa and other African nations, as they can use the Et-HEI with minor adaptations. For Ethiopia, the Et-HEI results will be used to establish a target for Ethiopian FBDG adherence, which will be relevant for designing, implementing and evaluating public health programmes and policies.

A limitation of our study is that the evaluation of the Et-HEI used only a population size of 494 women. Our results may thus have been different if the assessment was conducted in another population group which may influence our conclusion. Further research is advised to assess Et-HEI in various subpopulation groups and for a longer time based on the recommendations in the FBDG to use Et-HEI for individual nutrition counselling. However, based on our study, the Et-HEI can also be used to monitor and evaluate Ethiopian FBDG implementation's impact on the population at large because the Et-HEI was developed based on the FBDG recommendations for the general population of 2 years and older. In addition, in the Ethiopian FBDG, women of reproductive age were categorised as requiring medium amounts of energy which can be considered an average for the general population. Therefore, the Et-HEI can be used to assess the diet quality of the population by administering the Et-HEI at an individual level and reporting at the population level^(41)^.

## Conclusion

The Et-HEI we developed was able to indicate nutrient adequacy while determining adherence to FBDG. The total Et-HEI score was low, indicating the study population's consumption patterns had poor adherence to the 2022 Ethiopian FBDG. The low Et-HEI score was reflected by a low MDD-W score, a high score for only the plant-based components in the diet, and little diet diversity (almost zero fruits, nuts and seeds and a lack of animal-source foods). Poor adherence to the Ethiopian FBDG was underlined by a low probability of adequate vitamin A, vitamin B12, vitamin C and calcium intake. The Et-HEI may be a useful index to monitor and evaluate the implementation of the Ethiopian FBDG and other dietary interventions.

## Supplementary material

For supplementary material accompanying this paper visit https://doi.org/10.1017/jns.2022.120. click here to view supplementary material
