# Supplementary material for: Development of the Ethiopian Healthy Eating Index (Et-HEI) and evaluation in women of reproductive age
Source: J Nutr Sci. 2023 Jan 23;12:e9. doi: 10.1017/jns.2022.120 (PMC9879874; doi:10.1017/jns.2022.120)
Supplement: Supplementary file 1 [file S2048679022001203sup001.zip › S2048679022001203sup001.docx]

**Supplemental Table 1. Description of the Et-HEI components based on the Ethiopian FBDG**

| **No** | **Components** | **Description** |
| --- | --- | --- |
|  | ***Whole grains, roots, and tubers*** | Teff (Ethiopian cereal), potato, wheat, rice, maize, and sweet potato are part of this component. In the FBDG, grains, roots, and tubers are recommended to be consumed in combination with other food groups. Ultra-processed cereal products, such as cookies and potato chips, should be avoided in favour of roasted cereals (kollo) as a healthy snack. A daily intake of 400-650 g is recommended in the Ethiopian FBDG, and the amount of 570 g/day or more is given the maximum score. |
|  | ***Vegetables*** | Ethiopian kale, onion, green pepper, lettuce, carrot, tomato, head cabbage, and pumpkin are commonly consumed vegetables in Ethiopia. FBDG messages include choosing vegetables in season, eating various vegetables of different colours every day, and washing vegetables with clean water before eating them. Vegetables provide essential vitamins and minerals that protect from diseases. A daily intake of at least 130 g is recommended. |
|  | ***Fruits*** | Banana, papaya, lemon, watermelon, avocado, mango, pineapple, orange, and strawberry are common fruits in Ethiopia. It is suggested that fruits be washed with clean water before eating them, to choose seasonal and diverse fruits of different colours daily. Since eating more fruits lowers the risk of chronic diseases such as type 2 diabetes, at least 150 grams should be consumed daily. |
|  | ***Milk and dairy foods*** | Yogurt, milk, and cottage cheese are common dairy products in Ethiopia. The guidelines list dairy, meat, fish, and eggs as animal-sourced food. Every day, at least one meal containing dairy products is recommended. Furthermore, dairy products provide minerals such as calcium, essential for bone health. Milk and dairy foods should be consumed 200 to 400 grams daily. |
|  | ***Meat, fish, and egg*** | Chicken, fish, beef, eggs, and dried meat fall into this category. It is recommended to include animal-sourced foods in daily meals and replace them with legumes during fasting. They are good sources of protein, vitamins, and minerals. An average daily intake range from 20-50g/day is recommended. |
|  | ***Legumes*** | Beans, chickpeas, lentils, split peas, shiro, and peas are among the legumes mostly consumed in Ethiopia. Legumes are recommended to be added to kollo and nifro (a mix of boiled cereals and legumes). Different legumes should be added to the traditional Ethiopian stew (Shiro), made from various legumes, and mouldy legumes should be avoided. Consumption of legumes provides a good source of protein. At least 90 grams of legumes should be consumed per day. |
|  | ***Nuts and seeds*** | Niger, flaxseed, peanut, and sunflower seeds are examples of nuts and seeds consumed in Ethiopia. Nuts should be used the same way as legumes in the healthy snack called kollo (a mix of nuts and other cereals). Nuts and seeds are also recommended as a good source of protein, minerals, and unsaturated fat. Average daily consumption of at least 15 g is recommended. |
|  | ***Fats and oils*** | In Ethiopia, butter, meat fat, palm oil, sunflower oil, soy oil, and vegetable oil are common fat and oil sources. While using them sparingly, liquid vegetable oils such as sunflower, soya, and nut oils with relatively less saturated fat should be chosen. Furthermore, eating some fats and oils is advised to provide energy, help protect organs and keep the body warm. Fats and oils should be limited to 10–17 grams/day. |
|  | ***Added sugar and sugar-sweetened beverages (SSB)*** | Sugar, sweets, soft drinks such as leslasa, and packed juice should be consumed in moderation. According to the FBDG, sugar and sweeteners should also be avoided in milk, fruit juices, tea, and coffee. It is recommended to consume less than 30 g per day |
|  | ***Salt*** | Salt is recommended to be iodized and added after cooking (not during the process). The daily consumption of salt should be no more than 5 grams, according to the Ethiopian FBDG. |
|  | ***Alcohol*** | Alcoholic beverages such as tella, tej, beer, and areka should be consumed in moderation, with no more than 2 glasses per week. For pregnant or lactating women and children, alcohol consumption should be avoided. |
