# Supplementary material for: Development of the Ethiopian Healthy Eating Index (Et-HEI) and evaluation in women of reproductive age
Source: J Nutr Sci. 2023 Jan 23;12:e9. doi: 10.1017/jns.2022.120 (PMC9879874; doi:10.1017/jns.2022.120)
Supplement: Supplementary file 1 [file S2048679022001203sup001.zip › S2048679022001203sup002.docx]

**Supplemental Table 2**. Median percent of energy for carbohydrate, fat, and protein intake and the mount nutrient intake per 1800 kcal across Et-HEI quartiles

| Macro and micronutrient intake | | Quartiles of Et-HEI | | | | *p-value |
| --- | --- | --- | --- | --- | --- | --- |
|  |  | Q1  (n = 124) | Q2  (n= 123) | Q3  (n= 124) | Q4  (n= 123) |  |
| Carbohydrate (% energy) | | 73.6 | 75.2 | 76.1 | 76.2 | 0.020 |
| Protein (% energy) | | 9.94 | 10.1 | 10.2 | 10.5 | 0.046 |
| Fat (% energy) | | 16.3 | 14.5 | 13.7 | 13.6 | 0.036 |
| Micronutrients per 1800 kcal | |  |  |  |  |  |
|  | Vitamin A (RAE, µg/d) | 344.3 | 450.9 | 368.6 | 3.09.1 | 0.698 |
|  | Vitamin B6 (mg/d) | 1.51 | 1.52 | 1.51 | 1.50 | 0.814 |
|  | Vitamin B12 (ug/d) | 0.21 | 0.11 | 0.16 | 0.27 | 0.017 |
|  | Vitamin C (mg/d) | 27.8 | 27.0 | 25.4 | 23.2 | 0.108 |
|  | Calcium (mg/d) | 454.4 | 506.3 | 474.4 | 457.3 | 0.959 |
|  | Folate (ug/d) | 308.2 | 323.2 | 313.6 | 320.4 | 0.448 |
|  | Iron (mg/d) | 48.1 | 46.6 | 46.8 | 48.1 | 0.560 |
|  | Vitamin B3 (mg/d) | 9.76 | 9.49 | 9.47 | 9.20 | 0.048 |
|  | Vitamin B2 (mg/d) | 0.99 | 1.08 | 1.03 | 1.00 | 0.738 |
|  | Vitamin B1 (mg/d) | 0.93 | 0.96 | 1.01 | 1.04 | 0.203 |
|  | Zinc (mg/d) | 8.75 | 8.75 | 8.94 | 9.38 | 0.458 |
| * P-value from regression and estimated robust standard error for the association of nutrients intake per 1800 kcal across the Et-HEI quartile | | | | | | |
