# Supplementary material for: Development of the Ethiopian Healthy Eating Index (Et-HEI) and evaluation in women of reproductive age
Source: J Nutr Sci. 2023 Jan 23;12:e9. doi: 10.1017/jns.2022.120 (PMC9879874; doi:10.1017/jns.2022.120)
Supplement: Supplementary file 1 [file S2048679022001203sup001.zip › S2048679022001203sup003.docx]

**Supplemental Table 3**. The correlation between the 11 components and the total score of Et-HEI and total energy intake of women (N=494)

| **Et-HEI components** | **Whole grains, roots, and tubers** | **Vegetables** | **Fruits** | **Milk and dairy foods** | **Meat, fish, and egg** | **Legumes** | **Nuts and seeds** | **Fats and oils** | **Added sugar and SSB** | **Salt** | **Alcohol** | **Et-HEI total score** | **Energy intake** |
| --- | --- | --- | --- | --- | --- | --- | --- | --- | --- | --- | --- | --- | --- |
| Whole grains, roots, and tubers | 1.00 |  |  |  |  |  |  |  |  |  |  |  |  |
| Vegetables | 0.01 | 1.00 |  |  |  |  |  |  |  |  |  |  |  |
| Fruits | -0.01 | 0.07 | 1.00 |  |  |  |  |  |  |  |  |  |  |
| Milk and dairy foods | 0.10 | -0.03 | -0.02 | 1.00 |  |  |  |  |  |  |  |  |  |
| Meat, fish, and egg | -0.01 | -0.07 | 0.10* | 0.10* | 1.00 |  |  |  |  |  |  |  |  |
| Legumes | 0.06 | -0.20* | 0.06 | -0.013* | -0.07 | 1.00 |  |  |  |  |  |  |  |
| Nuts and seeds | 0.01 | -0.07 | -0.05 | -0.09 | -0.04 | 0.12 | 1.00 |  |  |  |  |  |  |
| Fats and oils | 0.10* | -0.14* | -0.10* | 0.04 | 0.01 | -0.07 | -0.04 | 1.00 |  |  |  |  |  |
| Added sugar and SSB | -0.00 | -0.09* | -0.17* | -0.03 | -0.05 | -0.05 | 0.02 | 0.17* | 1.00 |  |  |  |  |
| Salt | -0.05 | -0.46* | 0.04 | 0.01 | 0.05 | 0.00 | 0.02 | 0.17* | 0.04 | 1.00 |  |  |  |
| Alcohol | -0.15* | 0.34* | -0.02 | 0.02 | -0.10* | -0.25* | -0.24* | -0.04 | -0.06 | -0.30* | 1.00 |  |  |
| Et-HEI total score | 0.24* | 0.10* | 0.13* | 0.28* | 0.25* | 0.19* | 0.08 | 0.52* | 0.33* | 0.30* | 0.22* | 1.00 |  |
| Energy intake | 0.70* | 0.00 | 0.01 | 0.08 | -0.03 | 0.34* | 0.12* | -0.06 | -0.04 | -0.05* | -0.31* | 0.13 | 1.00 |
