# Supplementary material for: Development of the Ethiopian Healthy Eating Index (Et-HEI) and evaluation in women of reproductive age
Source: J Nutr Sci. 2023 Jan 23;12:e9. doi: 10.1017/jns.2022.120 (PMC9879874; doi:10.1017/jns.2022.120)
Supplement: Supplementary file 1 [file S2048679022001203sup001.zip › S2048679022001203sup004.docx]

**Supplemental Table 4**. Median, interquartile range indices, and the correlation of the components of Et-HEI with corresponding food groups of MDD-W and total score

| **Components** | **Et-HEI**  Median (P25, P75) | **MDD-W**  Median (P25, P75) | τ |
| --- | --- | --- | --- |
| Whole grains, roots, and tubers | 9.8 (7.4, 10) | 1 (1, 1) | 0.07 |
| Vegetables | 9.6 (4.5, 10) | NA | NA |
| Dark green leafy vegetables | NA | 0 (0, 1) | 0.43** |
| Vitamin A-rich vegetables and fruits | NA | 0 (0, 0) | 0.18** |
| Other vegetables | NA | 1 (1, 1) | 0.20** |
| Fruits | 0 (0, 0) | NA | NA |
| Vitamin A-rich vegetables and fruits | NA | 0 (0, 0) | 0.14* |
| Other fruit | NA | 0 (0, 0) | 0.91** |
| Milk and dairy foods | 0 (0, 0) | 0 (0, 0) | 0.94** |
| Meat, fish and eggs | 0 (0, 0) | NA | NA |
| Eggs | NA | 0 (0, 0) | 0.27** |
| Legumes | 5.2 (1.3, 10) | 1 (0.5, 1) | 0.62** |
| Nuts and seeds | 0 (0, 0) | 0 (0, 0) | 0.97** |
| Fats and oils | 5.8 (0, 9.4) | NA | NA |
| Added sugar and SSB | 10 (10, 10) | NA | NA |
| Salt | 0.3 (0, 10) | NA | NA |
| Alcohol | 10 (0, 10) | NA | NA |
| Total score^+^ | 49 (43, 54) | 3.5 (3, 4) | 0.30** |
| ^+^Spearman correlation is applied for the correlation between the total score of Et-HEI and MDD-W  *P-value<0.05 and **P-value<0.05 NA: Not Applicable | | | |
