# Supplementary material for: Development of the Ethiopian Healthy Eating Index (Et-HEI) and evaluation in women of reproductive age
Source: J Nutr Sci. 2023 Jan 23;12:e9. doi: 10.1017/jns.2022.120 (PMC9879874; doi:10.1017/jns.2022.120)
Supplement: Supplementary file 1 [file S2048679022001203sup001.zip › S2048679022001203sup005.pptx]

## Slide 1
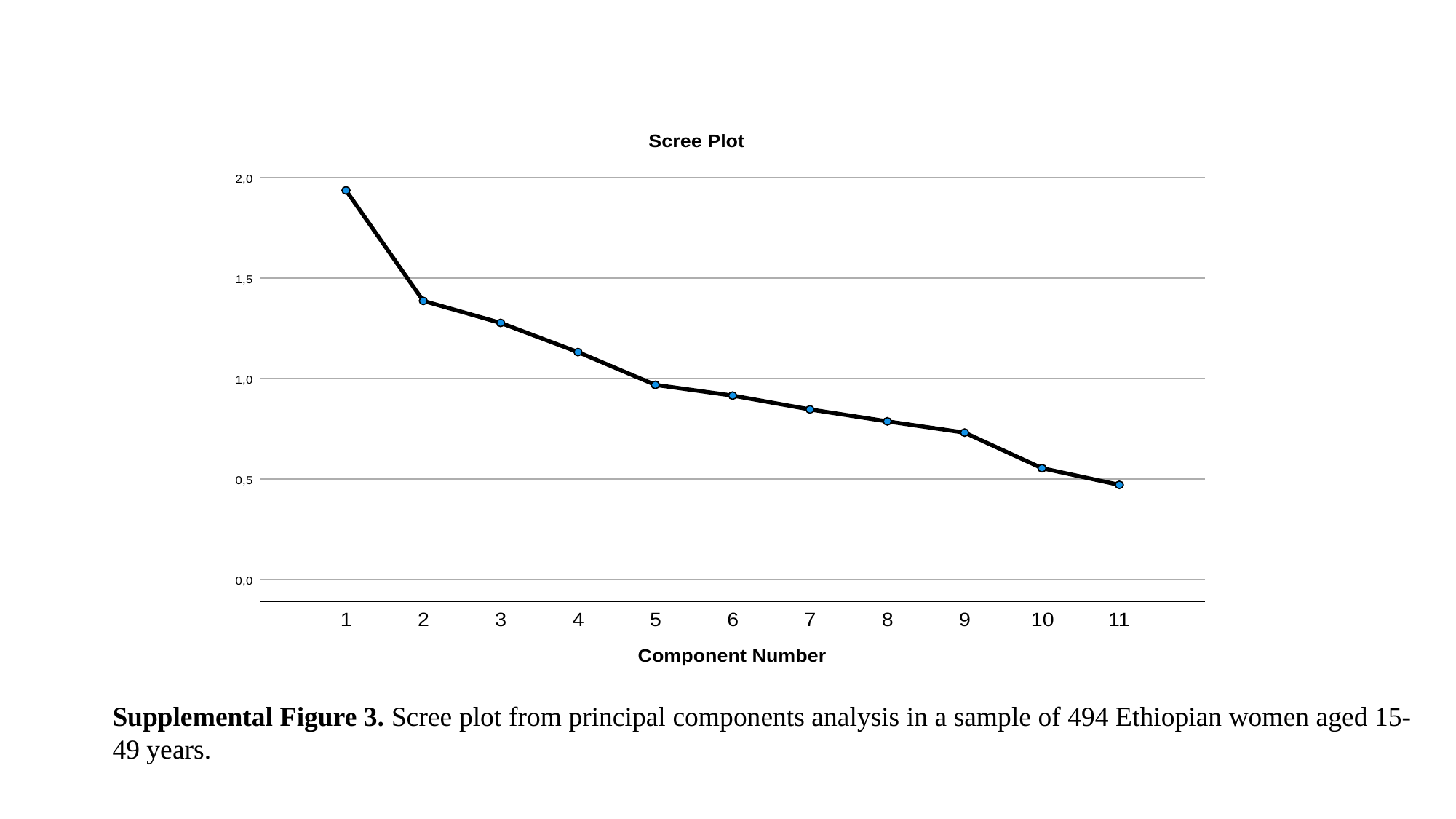

Supplemental Figure 3. Scree plot from principal components analysis in a sample of 494 Ethiopian women aged 15-49 years.
