# Supplementary material for: Development of the Ethiopian Healthy Eating Index (Et-HEI) and evaluation in women of reproductive age
Source: J Nutr Sci. 2023 Jan 23;12:e9. doi: 10.1017/jns.2022.120 (PMC9879874; doi:10.1017/jns.2022.120)
Supplement: Supplementary file 1 [file S2048679022001203sup001.zip › S2048679022001203sup006.pptx]

## Slide 1
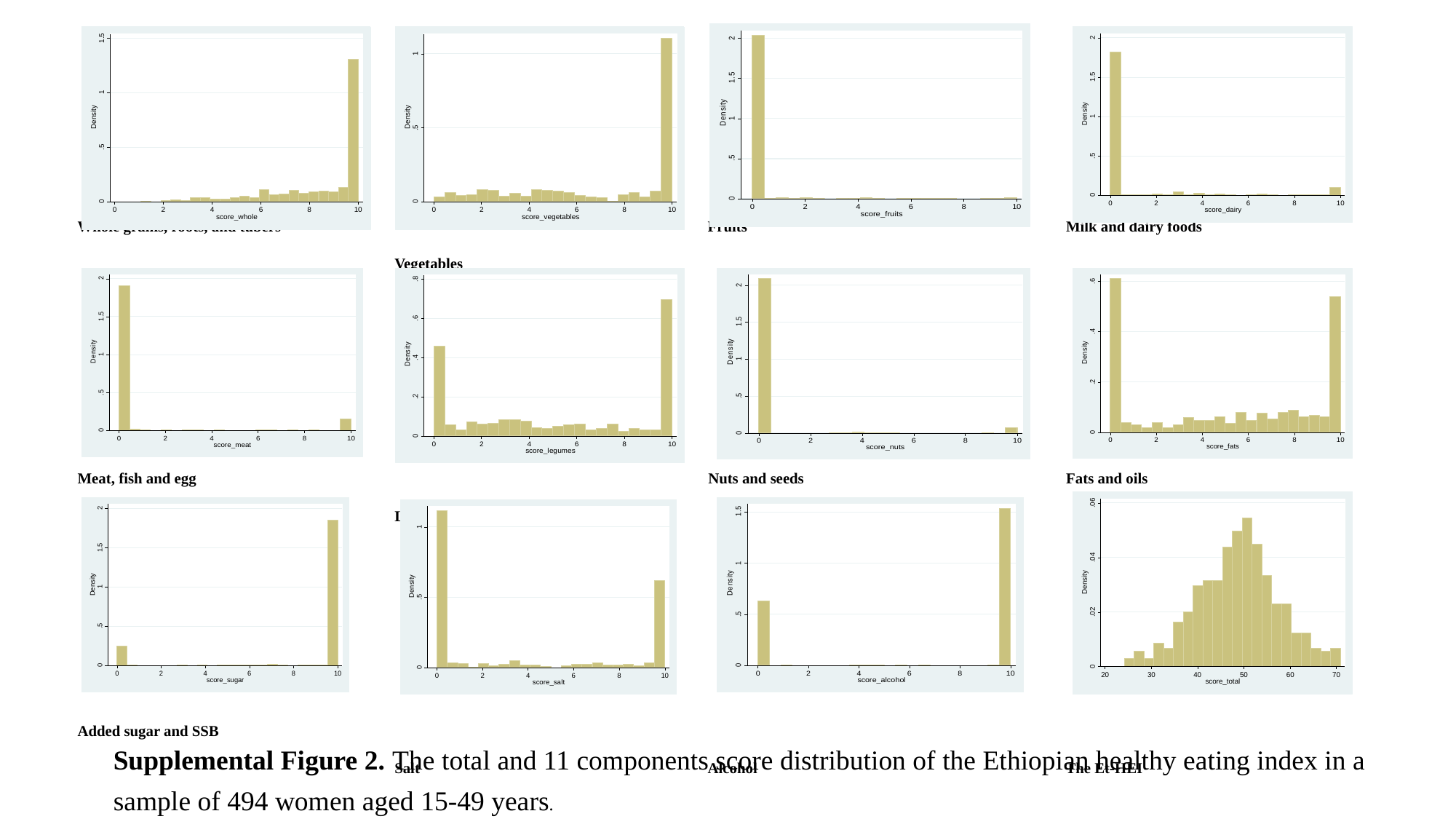

| Whole grains, roots, and tubers | Vegetables | Fruits | Milk and dairy foods |
| --- | --- | --- | --- |
| Meat, fish and egg | Legumes | Nuts and seeds | Fats and oils |
| Added sugar and SSB | Salt | Alcohol | The Et-HEI |
Supplemental Figure 2. The total and 11 components score distribution of the Ethiopian healthy eating index in a sample of 494 women aged 15-49 years.
